# Supplementary material for: Predicting Apoptosis Protein Subcellular Locations based on the Protein Overlapping Property Matrix and Tri-Gram Encoding
Source: Int J Mol Sci. 2019 May 11;20(9):2344. doi: 10.3390/ijms20092344 (PMC6539631; doi:10.3390/ijms20092344)
Supplement: Supplementary file 1 [file ijms-20-02344-s001.pdf]

Predicting Apoptosis Protein Subcellular Locations based on the Protein Overlapping Property Matrix and Tri-Gram Encoding

Table S1. Specificity comparison of different methods on the ZW225 dataset (%)

Methods	Cyto	Memb	Mito	Nucl
DF_SVM [5]	82.4	86.3	76.2	85.7
PSSM_AC [8]	88.4	95.6	99.0	94.6
Tri-gram PSSM [18]	98.1	98.5	100	100
PsePSSM-DCCA-LFDA [25]	99.4	100	100	100
Our method	98.1	100	100	99.5

Table S2. Specificity comparison of different methods on the CL317 dataset (%)

Methods	Cyto	Memb	Mito	Secr	Nucl	Endo
DF_SVM [5]	87.4	90.4	83.9	86.7	86.5	91.7
PSSM-AC [8]	94.6	98.1	98.2	100	97.7	100
Tri-gram PSSM [18]	97.6	98.9	98.9	99.7	99.6	100
PsePSSM-DCCA-LFDA [25]	100	100	99.6	100	100	100
Our method	98.5	98.9	99.3	100	99.2	99.3

Table S3. MCC comparison of different methods on the ZW225 dataset

Methods	Cyto	Memb	Mito	Nucl
DF_SVM [5]	0.78	0.82	0.66	0.75
PSSM_AC [8]	0.697	0.879	0.757	0.719
Tri-gram PSSM [18]	0.948	0.972	0.977	0.985
PsePSSM-DCCA-LFDA [25]	0.99	0.99	1	1

Our method	0.970	0.972	0.977	0.985

Table S4. MCC comparison of different methods on the CL317 dataset

**Methods**	**Cyto**	**Memb**	**Mito**	**Secr**	**Nucl**	**Endo**
DF_SVM [5]	0.84	0.85	0.77	0.80	0.84	0.91
PSSM-AC [8]	0.877	0.890	0.872	0.903	0.849	0.975
Tri-gram PSSM [18]	0.952	0.945	0.919	0.870	0.965	0.975
PsePSSM-DCCA-LFDA [25]	0.99	1	0.99	1	1	1
Our method	0.973	0.923	0.951	0.936	0.954	0.950
